# Supplementary material for: Neural Correlates of Impaired Cognitive Control in Individuals with Methamphetamine Dependence: An fMRI Study
Source: Brain Sci. 2023 Jan 24;13(2):197. doi: 10.3390/brainsci13020197 (PMC9954217; doi:10.3390/brainsci13020197)
Supplement: Supplementary file 1 [file brainsci-13-00197-s001.zip › brainsci-2139164-supplementary.pdf]

Supplementary Table S1. Maxima of regions showing significantly reduced fMRI signal in individuals with crystal meth dependence compared to healthy controls in the incongruent Stroop task condition (voxel-level p<0.001 uncorr., cluster size >16, according to the expected voxels per cluster)

Region of Activation	Left/Right	Brodmann Area	Cluster Size	MNI Coordinates x	y	z	t Value	$P_{FWE\text{-}corr}$
Occipital Cortex	R	17	3482	8	-73	16	5.82	0.000
Occipital Cortex	R	19	163	16	-61	-7	5.03	0.047
Superior Temporal Gyrus	R	22	296	54	-25	2	4.94	0.003
DLPFC	L	9	269	-27	40	38	4.74	0.005
Insula/VLPFC	R	13/44	174	44	4	6	4.06	0.037
Premotor Cortex	R	6	120	32	-13	54	4.70	0.132
Parahippocampal Gyrus	R	36	23	16	-37	-15	4.53	0.962
Occipital Cortex	L	19	305	-43	-65	2	4.49	0.002
Frontal Eye Field	R	8	56	18	30	56	4.48	0.602
Thalamus	R		42	12	-7	6	4.48	0.779
Motor Cortex	R	4	41	60	-13	46	4.45	0.792
Medial Temporal Gyrus	L	21	58	-69	-31	-13	4.41	0.578
Fusiform Gyrus	R	37	105	42	-35	-19	4.36	0.190
Premotor Cortex	L	6	63	-23	15	52	4.35	0.519
Supramarginal Gyrus	L	40	31	-45	-33	26	4.30	0.902
Somatosensory Cortex	L	1	99	-59	-15	32	4.29	0.220
Supramarginal Gyrus	L	40	97	-49	-39	56	4.27	0.232
aMCC	R	24	45	10	4	32	4.26	0.742
Premotor Cortex	L	6	22	-59	12	40	4.15	0.968
Superior Temporal Gyrus	L	22	32	-47	-41	14	4.11	0.892
Cerebellum	L		137	-25	-35	-47	4.10	0.087
Premotor Cortex	L	6	20	-35	-17	60	4.09	0.977
Dorsal PCC	R	31	23	10	-37	42	4.08	0.962
DLPFC	R	9	105	36	50	30	4.07	0.190
Angular Gyrus	L	39	92	-33	-49	34	4.06	0.262
Hypothalamus	R		30	2	-3	-13	4.05	0.911
DLPFC	R	9	52	38	32	30	4.03	0.652

Fusiform Gyrus	L	37	29	-17	-35	-25	3.96	0.920
Insula	L	13	37	-33	-9	12	3.96	0.839
Frontal Eye Field	R	8	44	40	32	46	3.95	0.754
Superior Parietal Lobule	L	7	44	-33	-61	46	3.95	0.754
Inferior Temporal Gyrus	L	20	48	-47	-9	-33	3.94	0.703
Putamen	L		33	-19	-5	-6	3.91	0.882
Premotor Cortex	R	6	23	44	-3	48	3.90	0.962
Insula	R	13	30	30	-15	14	3.88	0.911
Cerebellum	L		16	-15	-45	-53	3.88	0.990
DLPFC	L	46	65	-37	36	18	3.87	0.496
Premotor Area	L	6	36	-31	10	64	3.87	0.850
Angular Gyrus	R	39	45	62	-45	28	3.86	0.742
RLPFC	L	10	30	-25	56	18	3.76	0.911
Superior Parietal Lobule	L	5	40	-29	-43	56	3.76	0.804
Premotor Cortex	L	6	40	-5	-19	70	3.76	0.804
Fusiform Gyrus	L	37	21	-25	-29	-31	3.75	0.972
Caudate	L		28	-17	14	8	3.75	0.928
Frontal Eye Field	R	8	37	2	14	38	3.75	0.840
Motor Cortex	L	4	36	-59	-7	38	3.71	0.850
Cerebellum	R		90	24	-47	-51	3.71	0.275
Supramarginal Gyrus	R	40	16	68	-15	28	3.70	0.990
Thalamus	L		33	-15	-15	12	3.68	0.882
Superior Parietal Lobule	R	7	72	16	-75	50	3.64	0.423
Superior Parietal Lobule	R	7	37	16	-65	50	3.60	0.839
Supramarginal Gyrus	R	40	32	56	-29	48	3.60	0.892
DLPFC	R	46	19	44	34	8	3.52	0.981
Superior Parietal Lobule	L	5	18	-13	-31	48	3.50	0.984

***Abbreviations:** R, right; L, left; DLPFC, Dorsolateral Prefrontal Cortex; RLPFC, Rostrolateral Prefrontal Cortex; PCC, Posterior Cingulate Cortex; aMCC, Anterior Midcingulate Cortex; PCC, Posterior Cingulate Cortex.*

Supplementary Table S2. Maxima of regions showing significantly reduced fMRI Signal in individuals with crystal meth dependence compared to healthy controls in the congruent Stroop task conditions (voxel-level p<0.001 uncorr., cluster size >16, according to the expected voxels per cluster)

Region of Activation	Left/ Right	Brodmann Area	Cluster Size	MNI Coordinates			t Value	$P_{FWE\text{-}corr}$
				x	y	z		
Occipital Cortex	R	17	1744	8	-73	16	5.05	0.000
Somatosensory Cortex	R	1	16	60	-13	46	4.32	0.990
Superior Parietal Lobule	L	5	65	-29	-43	56	4.25	0.496
Superior Temporal Gyrus	R	22	146	48	-29	2	4.24	0.071
Medial Temporal Gyrus	L	21	25	-69	-35	-13	4.04	0.950
Ventral PCC	R	23	18	6	-17	30	4.03	0.984
Occipital Cortex	R	19	98	20	-75	30	3.96	0.226
Occipital Cortex	L	19	59	-47	-83	14	3.75	0.556
Angular Gyrus	R	39	28	32	-57	42	3.75	0.928
Occipital Cortex	L	19	82	-43	-67	2	3.74	0.334
Superior Parietal Lobule	R	7	55	16	-77	52	3.71	0.615
DLPFC	L	9	17	-27	38	38	3.68	0.987
Angular Gyrus	L	39	44	-35	-51	34	3.65	0.754
VLPFC	R	44	21	48	14	16	3.53	0.972
Angular Gyrus	L	39	28	-31	-75	26	3.50	0.928
Fusiform Gyrus	R	37	20	40	-45	-27	3.49	0.977

Abbreviations: *R, right; L, left; PCC, Posterior Cingulate Cortex; DLPFC, Dorsolateral Prefrontal Cortex; VLPFC, Ventrolateral Prefrontal Cortex.*

Supplementary Table S3. Maxima of regions showing significantly reduced fMRI Signal in individuals with crystal meth dependence compared to healthy controls in both Stroop task conditions together (voxel-level p<0.001 uncorr., cluster size >16, according to the expected voxels per cluster)

Region of Activation	Left/Right	Brodmann Area	Cluster Size	MNI Coordinates			t Value	$P_{FWE\text{-}corr}$
				x	y	z		
Occipital Cortex	L	17	3700	-8	-73	16	5.92	0.000
Superior Temporal Gyrus	R	22	320	48	-27	2	4.86	0.002
Somatosensory Cortex	R	1	41	60	-13	46	4.78	0.792
DLPFC	L	9	120	-27	40	38	4.52	0.132
VLPFC	R	44	180	50	4	18	3.87	0.032
Inferior Temporal Gyrus	L	20	50	-43	-31	-17	4.52	0.678

Occipital Cortex	R	19	21	16	-37	-15	4.47	0.972
SMA	R	6	83	32	-13	54	4.45	0.326
Fusiform Gyrus	R	37	136	-35	-55	-17	4.45	0.090
Occipital Cortex	L	19	375	-43	-67	2	4.44	0.001
Medial Temporal Gyrus	L	21	54	-69	-33	-13	4.40	0.627
SMA	L	6	45	-23	-13	50	4.39	0.742
Superior Parietal Lobule	L	5	75	-29	-43	56	4.37	0.394
Fusiform	R	37	134	42	-35	-21	4.29	0.094
Somatosensory Cortex	L	1	91	-49	-39	56	4.23	0.268
Superior Temporal Gyrus	L	22	35	-47	-41	14	4.18	0.861
Ventral PCC	R	23	22	6	-17	30	4.14	0.968
Angular Gyrus	L	39	119	-35	-49	34	4.13	0.135
Cerebellum	L		23	-15	-45	-53	4.11	0.962
Cerebellum	R		17	4	-35	-11	4.09	0.987
Frontal Eye Field	L	8	56	-41	28	48	4.08	0.602
Cerebellum	L		34	-27	-39	-49	4.07	0.872
Cerebellum	L		89	-31	-59	-53	4.03	0.282
Angular Gyrus	R	39	48	32	-57	42	3.98	0.703
Thalamus	R		21	12	-7	6	3.97	0.972
DLPFC	R	9	39	38	32	32	3.97	0.816
Motor Cortex	L	4	46	-61	-5	38	3.91	0.729
Superior Parietal Lobule	R	7	120	14	-77	52	3.89	0.123
aMCC	R	32	63	2	14	38	3.88	0.519
Hypothalamus	R		16	2	-1	-13	3.87	0.990
Frontal Eye Field	R	8	30	18	30	56	3.87	0.911
DLPFC	R	9	54	36	50	30	3.85	0.627
Cerebellum	R		68	24	-45	-53	3.85	0.464
Premotor Cortex	R	6	23	44	-5	48	3.85	0.962
Superior Parietal Lobule	L	7	44	-31	-61	44	3.84	0.754
Somatosensory Cortex	L	1	58	-57	-17	34	3.84	0.578
Dorsal PCC	R	31	16	10	-37	42	3.84	0.990
Angular Gyrus	R	39	65	46	-55	28	3.81	0.496
Angular Gyrus	R	39	72	60	-45	28	3.81	0.423
Somatosensory Cortex	R	1	28	66	-15	26	3.76	0.928
DLPFC	R	46	29	48	32	10	3.76	0.920

RLPFC	L	10	22	-39	44	28	3.75	0.968
Angular Gyrus	L	39	56	-29	-75	26	3.71	0.602
Superior Parietal Lobule	R	7	24	28	-65	32	3.70	0.956
Fusiform Gyrus	R	37	38	56	-71	-1	3.68	0.828
Frontal Eye Field	R	8	19	20	44	40	3.63	0.981
Supramarginal Gyrus	R	40	28	52	-33	54	3.62	0.928
Superior Parietal Lobule	L	7	25	-5	-59	46	3.56	0.950
Occipital Cortex	R	19	17	42	-69	12	3.48	0.987
Premotor Cortex	R	6	32	2	17	68	3.48	0.892
Fusiform Gyrus	R	37	21	56	-51	-1	3.45	0.972

Abbreviations: *R, right; L, left; SMA, Supplementary Motor Area; aMCC, Anterior Midcingulate Cortex; PCC, Posterior Cingulate Cortex; DLPFC, Dorsolateral Prefrontal Cortex; VLPFC, Ventrolateral Prefrontal Cortex; RLPFC, Rostrolateral Prefrontal Cortex*

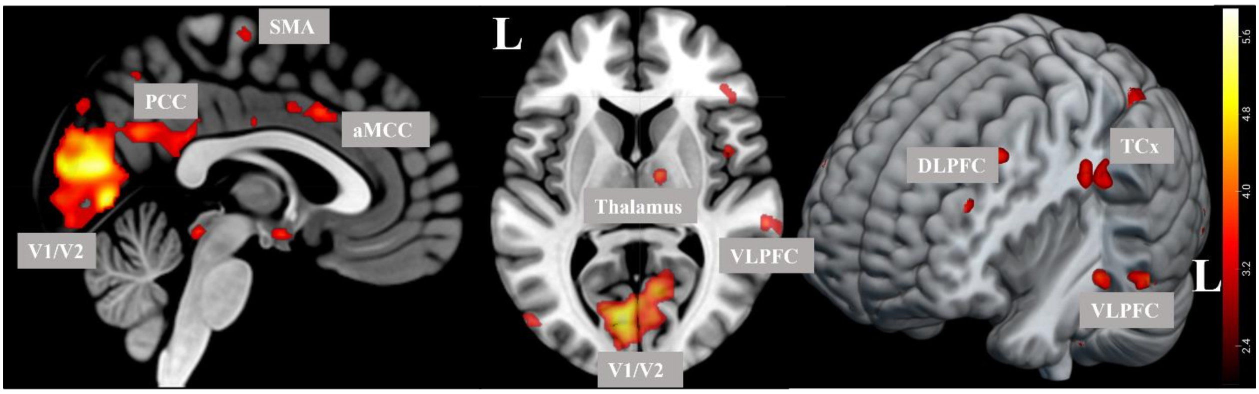

Supplementary Figure S1. *Significant overall group differences (healthy controls vs. individuals with crystal meth dependence) in brain activation during the Stroop task, both conditions together (voxel-level p<0.001 uncorr., cluster size >16,* according to the expected voxels per cluster*). Abbreviations: DLPFC, dorsolateral prefrontal cortex; VLPFC, ventrolateral prefrontal cortex; aMCC, anterior midcingulate cortex; V1, primary visual cortex; V2, secondary visual cortex; SMA, supplementary motor area; PCC, posterior cingulate cortex; TCx, temporal cortex*
